# Cooperative-Aware Radio Resource Allocation Scheme for 5G Network Slicing in Cloud Radio Access Networks

**DOI:** 10.3390/s23115111

**Published:** 2023-05-27

**Authors:** Salman A. AlQahtani

**Affiliations:** New Emerging Technologies and 5G Networks and Beyond Research Chair, Department of Computer Engineering, College of Computer and Information Sciences, King Saud University, P.O. Box 51178, Riyadh 11543, Saudi Arabia; salmanq@ksu.edu.sa

**Keywords:** cooperative communications, 5G network slicing, QoS, resource allocation, software-defined networking, network function virtualization

## Abstract

The 5G network is designed to serve three main use cases: enhanced mobile broadband (eMBB), massive machine-type communications (mMTC), and ultra-reliable and low-latency communications (uRLLC). There are many new technological enablers, including the cloud radio access network (C-RAN) and network slicing, that can support 5G and meet its requirements. The C-RAN combines both network virtualization and based band unit (BBU) centralization. Using the network slicing concept, the C-RAN BBU pool can be virtually sliced into three different slices. 5G slices require a number of Quality of service (QoS) metrics, such as average response time and resource utilization. In order to enhance the C-RAN BBUs utilization while protecting the minimum QoS of the coexisting three slices, a priority-based resource allocation with queuing model is proposed. The uRLLC is given the highest priority, while eMBB has a higher priority than mMTC services. The proposed model allows the eMBB and mMTC to be queued and the interrupted mMTC to be restored in its queue to increase its chance to reattempt the service later. The proposed model’s performance measures are defined and derived using a continuous-time Markov chain (CTMC) model and evaluated and compared using different methodologies. Based on the results, the proposed scheme can increase C-RAN resource utilization without degrading the QoS of the highest-priority uRLLC slice. Additionally, it can reduce the forced termination priority of the interrupted mMTC slice by allowing it to re-join its queue. Therefore, the comparison of the results shows that the proposed scheme outperforms the other states of the art in terms of improving the C-RAN utilization and enhancing the QoS of eMBB and mMTC slices without degrading the QoS of the highest priority use case.

## 1. Introduction

Presently, there is a continually increasing demand for higher data rates, better energy efficiency, better connection reliability regardless of location, and lower network latency. The fifth wireless generation (5G), which is expected to offer higher data rates and lower latency as well as meet other technical expectations, represents a massive jump in quality over previous generations [1,2,3,4]. The International Telecommunication Union (ITU) has defined the vision, requirements, and use cases of 5G networks. International Mobile Telecommunication 2020 Standards (IMT-2020) is the ITU standard that is used as a name for the components, systems, and other related elements designed to enable network capabilities beyond those offered by previous mobile wireless generations. The main role of IMT-2020 is to set 5G research activities and define the overall objectives of 5G standardization.

In order to meet the diverse requirements and demands for future network usage, the ITU has defined the important 5G use cases and classified them into three important categories. These categories are enhanced mobile broadband (eMBB), massive machine-type communications (mMTC), and ultra-reliable and low-latency communications (uRLLC) [5]. The main features of these categories (use cases) are as follows:

eMBB features human-dependent use cases that cover high broadband communication, enhancing access to multimedia such as ultra-high-definition (3D/UHD) video and augmented reality, transmitting very high-performance data, and handling the data rate explosion [4,5].

The mMTC is explicitly for the connection requests created by a huge number of small connected devices, such as the Internet of Things (IoT). These devices send and receive a low amount of non-delay-sensitive data and are required to have very low power consumption, low data rates, and reliability [6,7].

uRLLC is a use case that handles an ultra-responsive connection with ultra-low latency. This use case is well suited for handling sensitive applications, such as smart vehicles, remote medical surgery, safety, and mission-critical applications [8,9]. Table 1 shows the QoS requirements for each 5G use case and their priorities. In the table, priority (1) is the highest and priority (3) is the lowest. The table also presents the latency data rate. At the low end, mMTC has the lowest priority and has long latency, e.g., in a smart city [10].

In order for 5G networks to meet the QoS requirements for all of their use cases, many new technological features and enablers must be developed to support them. Agility and flexibility are the main defining features of 5G networks that enable them to meet the required ITU objectives. In order to meet the end-to-end flexibility requirements for 5G networks, network softwarization is defined as the main technology enabler for 5G networks. Network softwarization is a set of new technologies that separate the functions of the 5G core network hardware and software. This enabler technology of the 5G network includes software-defined networking (SDN), network function virtualization (NFV), cloud radio access network (C-RAN), and 5G network slicing.

SDN is a networking technology in which the control plane, logically centralized in a software-based controller network brain, is decoupled from the forwarding plane. SDN has many benefits, such as the acceleration of innovation and rapid adaptation to the demands and customization of network resources. It also promises to improve the programmability and flexibility of networks.

NFV aims to make a network more flexible and simpler by minimizing dependence on hardware constraints. NFV mainly decouples network functions from proprietary devices, such as firewalls, routers, and traffic controls, and runs their functions as software in virtual machines. This virtualization scenario will consolidate the many specialized types of network equipment used in the industry, reduce the cost of network changes and upgrades, and increase network efficiency and flexibility [2,3,4].

The 5G network slicing is a promising technology enabled by SDN and NFV for providing network service convergence and on-demand services tailored to users’ specific QoS requirements with limited network resources. It also reduces capital expenditures and operational expenses [5,8]. A slicing network can enhance the efficiency of the entire network and provide miscellaneous 5G services by utilizing a virtual network. Network slicing can divide the 5G physical network into many logical networks to support on-demand services for all expected requests and applications. It should be noted that the logical networks are in the same physical network and point to network slices [2,3,4,5,8,9].

The C-RAN is defined as one of the main components of the new architecture of 5G networks. Generally, the main radio resources located at the 5G base station (BS) include the radio antennas, the remote radio heads (RRHs), and the baseband units (BBUs). In order to improve the 5G architecture, the Next Generation Mobile Networks project transferred the BBUs from the BS to the 5G C-RAN. Therefore, all the BBUs of the RRHs connected by the same C-RAN are transferred and grouped into a virtualized BBU pool located in the C-RAN. In this new architecture, the BS is connected to the C-RAN using a high speed fronthaul connection and has only the RRHs and the radio [11,12,13]. With this new 5G C-RAN, the processing of the resource allocation is centralized at the C-RAN and can serve hundreds of distributed RRHs [3]. The BBUs are responsible for allocating the C-RAN resources to RRHs based on the defined network needs.

CRAN has three main components, which are BBU, RRU, and front-haul. This architecture is used in 5G, where it can serve as a transport network for 5G and improve network performance. It also has the ability to pool resources, simplify network management and operation, reduce energy consumption, and co-execute RAN functionalities along with other network functions in a data center. Distributed RAN is a classical setup where remote radio units and BBUs are collocated at every cell site, and it is not appropriate for enhancing network performance.

**Figure 1:**
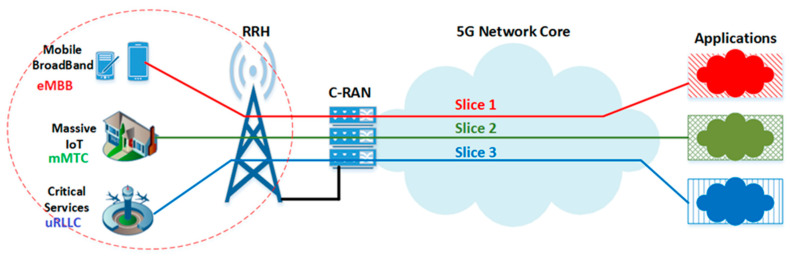
5G network slicing concept.

C-RAN combines both network virtualization and centralization of processing units (BBUs) in order to increase resource utilization and reduce the cost of deploying dense mobile networks. Using the network slicing concept, the C-RAN BBU pool can be virtually sliced into different slices, where each slice is allocated for different types of 5G use cases. These centralized and sliced resources are in critical need of efficient and flexible radio resource management [3]. Based on the 5G use cases, the BBU pool can be sliced into three slices, as shown in Figure 1. These 5G slices have different QoS requirements and different priority levels.

The 5G network is the future of mobile networks, and C-RAN is the 5G network component that groups all BBUs in a centralized BBU pool. In order to enhance C-RAN resource utilization while protecting the minimum QoS required for each 5G slice, an efficient radio resource allocation method is necessary and critical to controlling the coexisting 5G slices. This motivated us to propose a priority-based radio resource allocation method for 5G C-RAN with different coexisting 5G network slices. This method differentiates between the different slices based on their required QoS. The queuing model is used to store the delay-tolerant slices. This will decrease their forced termination rate and increase C-RAN resource utilization without degrading the QoS of the higher-priority slices. The proposed model allows the interrupted slice with the lowest priority to be restored in its queue in order to provide a chance to reattempt the service later. Therefore, it will increase its service completion rate without degrading the higher-priority slices. The performance measures of the proposed model are conducted and derived using a continuous-time Markov chain (CTMC) model.

The proposed scheme distinguishes the 5G use cases (eMBB, mMTC, and uRLLC) based on their priority level and uses a queuing system with a feedback queue to accommodate the arriving 5G slice requests with lower priority (eMBB, mMTC) or interrupted mMTC services. The queuing system with feedback will improve the service completion rate of eMBB and mMTC without degrading the uRLLC services, thereby increasing the BBU pool utilization of C-RAN. The contributions that distinguish this research from most of the previous works are as follows:

- A priority-based resource allocation scheme with a queuing model is proposed for 5G C-RAN with eMBB/mMTC/uRLLC coexistence.
- The priority queuing model is used to buffer the lower priority slices, and the interrupted non-delay-sensitive mMTC services are allowed to be returned to their queue to reattempt the service.
- Mathematical equations are derived for the main performance measures of the proposed scheme using a continuous-time Markov chain (CTMC) model.
- A simulation model is developed to compare with and validate the results of the proposed analytical model.
- The proposed system is compared with the traditional queuing system to verify its main objectives.

The remainder of this paper is organized as follows: In Section 2, related works are described. The system model and assumptions are explained in Section 3. In Section 4, the proposed work is explained. Furthermore, Section 5 provides the performance analysis of the proposed scheme. In Section 6, we provide the numerical results of the proposed scheme. Finally, concluding remarks are made in Section 7.

## 2. Related Works

The radio resource management and allocation methods in 5G networks with SDN/NFV-based functions were presented and studied in many research papers [8,9,11]. Several research efforts have introduced and analyzed the radio resource control and QoS provisions for 5G network slices [10,12,13,14,15,16].

The resource allocation strategies and challenges for network slicing were introduced and discussed in some recent works [8,9,11]. A survey of principles and a model for network slicing resource management were discussed in [8]. In [9], the problem of sharing 5G resources between different network slices in a fair manner was studied. In [11], network slicing in virtualized wireless networks was studied. Furthermore, a resource allocation algorithm was proposed for orthogonal frequency division multiple access (OFDMA) virtual RAN that can enhance the spectral efficiency of eMBB and uRLLC slices and improve uRLLC reliability.

In [12], three scheduling schemes for SDN-based network slicing were proposed to enhance the joint resource management problem with different QoS requirements. In [13], in order to achieve resource fairness and high utilization in the 5G network, packet delay modeling was proposed for the traffic flow through the chain of virtual network functions (VNFs). In [14], a 5G network slicing framework was presented in order to enable efficient isolation between the coexistence of IoT and eMBB slices.

In [10], a radio resource allocation method for network slicing is discussed. Specifically, a dynamic network resource allocation model for various 5G slices across different 5G network domains was suggested. As there are different requirements for each 5G slice in C-RAN, the proposed resource allocation aims to increase the level of satisfaction for each traffic slice in C-RAN and in all 5G network segments. In [15], the authors defined a new slicing architecture for the 5G network, as they considered it the main task to make 5G easier to understand. In addition, they presented a model for managing the constraints due to spectrum sharing by different access networks.

In [16], the end-to-end behavior of slice isolation is examined, especially from a security perspective. Isolations were classified as traffic, bandwidth, processing, or storage. A set of challenges for the recent trends in 5G slice isolation was discussed. The authors argued that isolation is the most important property of network slicing.

The authors of [6,17] provided a model that can work with different types of data. From this point on, different data types require a traffic scheduling technique, as this would make the QoS in 5G mobile networks easier to manage. The techniques they have suggested are priority queuing, first-in-first-out (FIFO), and weighted fair queuing. In [17], a queue model was assumed to interact with two packet types (real-time and non-real-time). Each packet type has its own transmission time, during which a scheduler allocates the packet to a resource unit (BBU). The queue model assigns a higher priority to real-time packets, and the higher priority will pass to the queue model via frequency feedback for packet transmission.

In [18], an efficient and secure service-oriented authentication framework that could support both network slicing and fog computing for the 5G-enabled Internet of Things network is proposed. In [19], a dynamic network slicing framework is presented in which a regional orchestrator is introduced to coordinate workload distribution among local fog nodes. In this node, the number of resources allocated to each slice could be dynamically adjusted according to service requests and energy availability. In [20], the authors proposed a network slicing architecture that utilizes spectrum resources in both licensed and unlicensed bands. In [21], an optimization framework is proposed that could jointly allocate resources for slices in terms of both network bandwidth and cloud processing power at a fine-grained level.

In [22,23], the concept of the queuing model was used to model the resource allocation problem for the coexistence of different services with different QoS in 4G or 5G networks. In [22,23], the coexistence of machine-to-machine (M2M) and human-to-human (H2H) communications in 4G networks was studied. In [22], a queuing model was used to analyze the impacts of coexistence between M2M and H2H communication on a 3GPP LTE system. In [23], a resource sharing method was proposed for M2M and H2H traffic under a time-controlled scheduling scheme in LTE networks.

In [24], the resource allocation problem for 5G network use cases across its main domains is analyzed using a general queuing model. This proposed scheme consists of three sub-queuing models covering the three main domains of the 5G network, which are C-RAN, mobile edge computing (MEC), and cloud data centers (CDCs). More specifically, the model aims to estimate the required QoS for network use cases in each component of the 5G architecture. In [25], a radio resource allocation for 5G use cases at 5G C-RAN is proposed using polling system methods. The aim of this scheme is mainly to protect the required delay for each 5G slice (use case) [26,27].

In [28], the authors proposed an allocation method that allocates the cost of the network to each deployed 5G network slice. This resource allocation method saves the core network costs. However, the QoS for each 5G slice was not addressed. In [29], an energy-efficient-based scheduling algorithm for eMBB and URLLC is proposed. The proposed algorithm is implemented as a mixed-integer non-linear problem. The numerical results proved that it outperformed the existing classical schemes. In [30], the impact of the SDN controller on packet delay is studied using an analytical mode. The study focused on two services: eMBB and mMTC. The results show that the proposed model can be used to plan the resource allocation method in 5G edge clouds in order to meet the QoS requirements for all 5G slices [31,32,33].

To the best of my knowledge, the balance between the provision of higher resource utilization in 5G C-RAN and the protection of QoS required by each of the coexisting slices has not been investigated. Therefore, we are motivated to investigate this issue by proposing a priority-based resource allocation that can accommodate the coexistence of 5G slices at the C-RAN node. Protecting slice isolation and ensuring maximum C-RAN utilization are the main goals of the proposed scheme.

## 3. System Model and Assumptions

**Figure 2:**
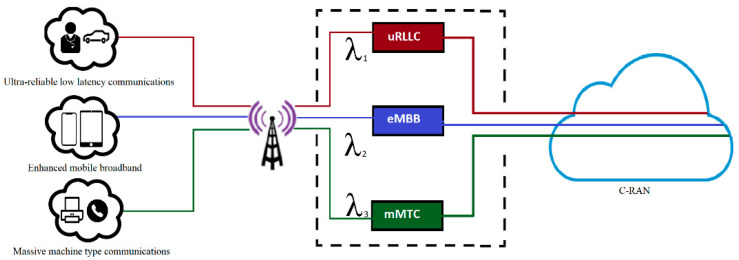
System model.

A single 5G C-RAN that has a BBU pool has been considered. Many RRHs are connected to the C-RAN through high-speed connections. In this paper, a single-cell area served by a single RRH through a 5G air interface is assumed. Each RRH serves many users, and each user’s request is related to one of the specified 5G slices. The C-RAN is assumed to have a C number of BBUs and is virtually sliced into three slices, where each slice is isolated for a specific service type. The general components of the proposed system are shown in Figure 2. The first slice is dedicated to uRLLC services, while the second slice is dedicated to eMBB services, and finally, the third slice is dedicated to mMTC services. All the RRH connection requests are sent to the BBU pool and classified based on their slices. The arrival rate of each request type is assumed to follow a Poisson distribution and is denoted as λ_1_, λ_2_, and λ_3_ for uRLLC, eMBB, and mMTC, respectively. The service time for all connection requests is assumed to be exponentially distributed with rates μ_1_, μ_2_, and μ_3_ for uRLLC, eMBB, and mMTC users, respectively. We use the variable C to represent the number of BBUs in the C-RAN BBU pool. The value of C will depend on the 5G bandwidth selected and is assumed to be statically identical. The connection is assumed to be millimeter wave with Giga speed.

The uRLLC (e.g., with a smart drive) has the highest priority, and the mMTC has the lowest priority (e.g., smart city applications). eMBB has a lower priority than the uRLLC (e.g., in smartphones). The arrival of an uRLLC can interrupt any in-service lower-priority requests if all BBUs are busy. In addition, the arrival of eMBB can interrupt any in-service mMTC if all BBUs are busy. The proposed model has two queues with finite lengths for eMBB and mMTC requests. The queued requests can wait until the resources are available.

## 4. Proposed Resource Allocation Scheme

**Figure 3:**
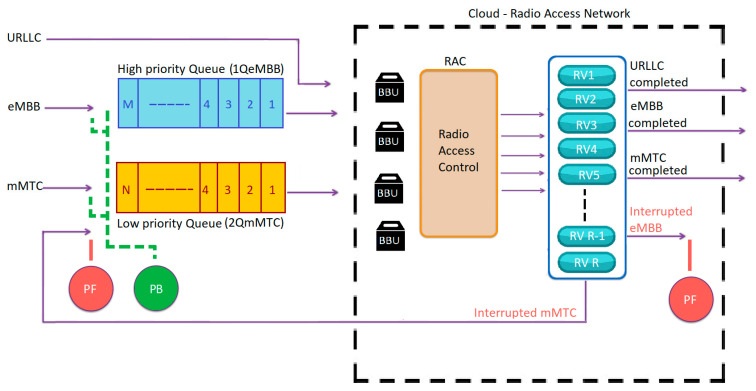
Proposed queuing model for the 5G slicing categories (1–4… N: queue size).

We propose a priority-based resource allocation method that resides in the C-RAN and has two queues. Two queues are provided, where the first is given to eMBB services and the second is allocated to mMTC services. Figure 3 shows the proposed scheme with the two queues. The incoming eMBB request is stored in its queue when the system is fully occupied and is indicated as 1QeMBB, whereas the incoming mMTC request is stored in its queue when all resources are busy and is called 2QmMTC. The sizes of 1QeMBB and 2QmMTC are assumed to be M and N, respectively. When we apply FIFO in the C-RAN, the C-RAN will allocate resources for each queue based on FIFO discipline. All BBUs are open for all incoming services, and 1QeMBB is served before 2QmMTC.

The main expected behaviors of the proposed scheme can be explained based on the occurrences of the following events:

- *uRLLC arrival*: when an arrival request from uRLLC occurs, it will go immediately to the BBU pool, and the BBUs will be checked first. If there are available BBUs, then the request is allocated to the required resources. If no BBUs are available, then we will have the following scenarios: If the same service type (uRLLC) occupies all BBUs of the C-RAN, then the received request is blocked. If the BBUs are fully occupied and there is at least one service type of eMBB or mMTC in progress, then the ongoing service type will be forced to terminate its occupied BBUs. Since eMBB has a higher priority than mMTC, the ongoing mMTC service will be interrupted first. If the interrupted service is of type mMTC, then it will be sent back to its queue if it is not full; otherwise, it will be dropped and considered a forced termination. If there is no ongoing mMTC, then the ongoing eMBB is forced to terminate its occupied BBUs.
- *An eMBB or mMTC arrival*: If the new arrival request is of type eMBB or mMTC, then if there is at least one available BBU resource, it will be allocated to the available resource. In the event that there are no available BBU resources, the arrival request will be queued in its corresponding queue. If its queue is full, then the new arrival request will be blocked.
- *An uRLLC, eMBB, or mMTC departure*: If any of the use cases complete their service and release the used BBU resources, then the resource becomes available. After that, the system will check the 1QeMBB queue, and if there is any waiting request, it will be allocated to the available resource based on FIFO discipline. If the 1QeMBB queue is empty, then the 2QmMTC queue will be checked and serviced if it has a queued request. If all queues are empty, then the available BBU will be left empty in the BBU pool.

The pseudocode for the main arrival process of the proposed scheme is shown in Table 2.

In order to study the performance of the proposed scheme and to compare it with related previous models, we will study its behavior using different processing methodologies, as follows:

- Methodology I: the resource allocation scheme has no queues, which is similar to the behaviors studied in [22,23,24].
- Methodology II: the resource allocation scheme has two queues for eMBB and mMTC without allowing the interrupted mMTC to rejoin its queue again.
- Methodology III (proposed scheme): the resource allocation scheme is similar to Methodology II, but it allows the interrupted mMTC to rejoin its queue again if it is not full. The arrival request of type uRLLC can preempt the lower-priority services that are in service.

## 5. Performance Analysis

The performance analysis of the proposed resource allocation scheme is explained in this section by using continuous-time Markov chains (CTMC) with multiple dimensions.

### 5.1. Analytical Model

In order to analyze and derive the performance measures of the proposed system, we define an analytical model using the continuous-time Markov chain (CTMC) model. The CTMC is used as a tool to model the proposed scheme and evaluate the scenarios being discussed. The proposed scheme is assumed to operate inside the 5G C-RAN component and manage the resource allocation for three different 5G network slices, namely the uRLLC, eMBB, and mMTC slices. The used CTMC model has five dimensions with variables (*i*, *j*, *k*, *m*, and *n*) and is based on organizing the requests of each 5G slice on a priority basis. These five variables represent the state of the proposed system at any given instance, where *i*, *j*, and *k* represent the number of ongoing uRLLC services, eMBB services, and mMTC services, respectively. The variables “*m*” and “*n*” correspond to the current number of queued services of type eMBB and mMTC request, respectively. The number of queued mMTC services includes the interrupted mMTC service requests that were forced to terminate their connections by the arrival of uRLLC requests.

The total current requests in the system (C-RAN) at any given time, including the queued requests, is the sum of *i* + *j* + *k* + *m* + *n*. Let us represent this total as T such that T = *i* + *j* + *k* + *m* + *n* and 0 ≤ T ≤ (C + M + N). Therefore, the state of the system can be represented by a five-dimensional state (i, j, k, m, and n), and when a new event occurs, the system will change its current state to another state, and the value of the five variables will change when the system state moves into different states. In order to derive the detailed balance equation of the proposed system and simplify its expression, we will define the variables, notations, and indicators that are used in the analytical model as follows:

The variable M represents the buffer size for storing new delay-sensitive eMBB requests. The variable N represents the second buffer, and this buffer has a size for storing new and interrupted-delay-tolerant mMTC requests. The variables M and N correspond to the maximum occupancy of 1QeMBB and 2QmMTC queues, respectively. The variables λ_1_, λ_2_, and λ_3_ are used to indicate the arrival rates of uRLLC, eMBB, and mMTC requests, respectively. Moreover, μ_1_, μ_2_, and μ_3_ are used to indicate the departure rates of uRLLC, eMBB, and mMTC requests, respectively. The 5G C-RAN is assumed to have C resource units (BBUs). Table 3 summarizes the main notations and parameter descriptions that are used here.

From the above discussion, we can represent the system state space, S, as:

(1) S={(i,j,k,m,n)|i≥0, j≥0, i+j+k≤C, 0≤m≤M, 0≤n≤N}

In the proposed system, there are two queues, and based on the queues’ status and the total number of free resources, we can classify the system state space (S) of the proposed system into different substates. For those substates, every single substate defines a section of the practical state of the proposed system. Furthermore, we must study the behavior of every single substate and define its balance equation. Hereafter, the detailed balance equation is derived, where the usage of several derived states leads us into the required balance equations. The queuing analysis methods presented in [26,27] were followed. In order to derive the proper balance equation for each substate, we need to define the number of Boolean indicators to control the transition rates between substates, as shown in Table 4.

The system state space represents the complete, feasible states of the proposed system. In order to simplify the analysis of the system, the system state space S can be described by using four substates S_i_ (*i* = 1, 2, 3, 4) such that S = $S_{1}\cup S_{2}\cup S_{3}\cup S_{4}$ and these substates can be defied as follows:

- S_1_ = {(i, j, k, m, n)| i = 0; j = 0; k = 0; m = 0; n = 0}
- S_2_ = {(i, j, k, m, n)|0 < (i + j + k) < C; m = 0; n = 0}
- S_3_ = {(i, j, k, m, n) | 0 < (i + j + k) = C; m = 0; n = 0}
- S_4_ = {(i, j, k, m, n)|C < (i + j + k + n + m) ≤ (C + M + N); 0 ≤ m ≤ M; 0 ≤ n ≤ N}.

In the following subsections, we briefly describe each single substates and derive their balance equation as follows.

Substate 1 (S_1_): These substates represent all the possible states where the whole system is empty, i.e., T=zero. That is, all of the resource units (BBUs) of C-RAN are free with no service. We call this an initial state. Therefore, the state space of S_1_ can be written as:

*S*_1_ = {(*i*, *j*, *k*, *m*, *n*): *i* = 0; *j* = 0; *k* = 0; *m* = 0; *n* = 0}

where all five variables are equal to zero and its balance equation can be defined as:

(2) $${(\lambda }_{1}+\lambda_{2}+\lambda_{3})\pi_{0,0,0,0,0}=\pi_{1,0,0,0,0}+\pi_{0,1,0,0,0}+\pi_{0,0,1,0,0}$$

Substate 2 (S_2_): These substates represent all the possible states (0 < T < C and m = n = 0) where the total number of requests in the system is greater than zero and less than C without any queued requests. Therefore, these substates show only the number of occupied BBUs at the current moment. Therefore, the total available BBU resources will be greater than zero and less than C when both queues are free or empty. Hence, the state space of S_2_ can be written as:

*S*_2_ = {(*i*, *j*, *k*, *m*, *n*)/0 < *i* + *j* + *k* < *C*; *m* = 0; *n* = 0} (2a)

Considering the possible states of S_2_, the detailed balanced equation for S_2_ can be represented as:

(3) $$\left(i\mu_{1}+\lambda_{1}+j\mu_{2}+\lambda_{2}+k\mu_{3}+\lambda_{3}\right)\pi_{i,j,k,0,0}=\lambda_{1}\pi_{i-1,j,k,0,0}+\left(i+1\right)\mu_{1}\pi_{i+1,j,k,0,0}+\lambda_{2}\pi_{i,j-1,k,0,0}+\left(j+1\right)\mu_{2}\pi_{i,j+1,k,0,0}+\lambda_{3}\pi_{i,j,k-1,0,0}+\left(k+1\right)\mu_{3}\pi_{i,j,k+1,0,0}$$

Substate 3 (S_3_): These substates represent the system states when *i* + *j* + *k* = C and m = *n* = 0. In this case, the whole system resource BBUs at this moment are fully utilized, while both queues 1QeMBB and 2QmMTC are still empty. Thus, the balanced equation for S_3_ can be represented as:

(4) $$\left(i\mu_{1}+\alpha_{1}\lambda_{1}+j\mu_{2}+\lambda_{2}+k\mu_{3}+\lambda_{3}\right)\pi_{i,j,k,0,0}=\alpha_{3}\lambda_{1}\pi_{i-1,j,k,0,0}+\left(j\mu_{2}+k\mu_{3}\right)\pi_{i,j,k,1,0}+\lambda_{2}\pi_{i,j-1,k,0,0}+k\mu_{3}\pi_{i,j,k,0,1}+\lambda_{3}\pi_{i,j,k-1,0,0}$$

Substates 4 (S_4_): These substates represent all the remaining feasible states of the system when 0 < T < C where 0 < m < = M and 0 < n < = N. In this case, clearly all BBUs are occupied, and the arrival of both eMBB and mMTC services has to wait in their own queues if the corresponding queue is not full. Thus, the balanced equation for S_3_ can be represented as:

(5) $$\left(\begin{array}{c} \alpha_{6}{i\mu }_{1}+\alpha_{1}\lambda_{1}+\alpha_{7}\left(1-\alpha_{6}\right){i\mu }_{1}+\\ {{\alpha_{6}j\mu_{2}+\alpha }_{7}\left(1-\alpha_{6}\right)j\mu }_{2}+\alpha_{8}k\lambda_{1}+\\ \alpha_{6}k\mu_{3}+{\alpha_{7}\left(1-\alpha_{6}\right)k\mu }_{3}+\alpha_{9}\lambda_{3} \end{array}\right)\pi_{i,j,k,m,n}=\alpha_{3}\lambda_{1}\pi_{i-1,j+1,k,m-1,n}+\alpha_{3}\lambda_{1}\pi_{i-1,j+1,k,m,n-1}+\alpha_{3}\lambda_{1}\pi_{i,j,k,m-1,n}+\alpha_{3}\lambda_{1}\pi_{i,j,k,m,n-1}$$

Using all the above substates and assuming that all other remaining states that are not covered by those substates are empty, we can derive the steady state probability of the system [23,24]. We assume that the Markov chain is memoryless and has a finite number of states. Within this memoryless feature, the constraint is that the work is performed at this moment or the current time (now), which is aperiodic, so it does not repeat itself. The Markov chain process can be described by an individual, time-independent matrix π_i,j,k,m,n_. In that regard, π_i,j,k,m,n_ will be assumed to be zero for non-feasible states when the steady-state probability is π_i,j,k,m,n_. Therefore, we define the value of the matrix π_i,j,k,m,n_ = 0 for the situation where (*i*,*j*,*k*,*m*,*n*) ∉ S.

Accordingly, having the defined system state, S, the transition matrix **Q**, and the vector π representing the steady-state probability satisfied the following condition:

(5a) $$\sum_{\begin{array}{c} \forall S\\ 0<\pi_{i,j,k,m,n}<1 \end{array}}\pi =1$$

Based on this condition, the system global balance equations and the normalization equation can be derived using the following two equations:

(6) $$\pi Q=0;\;and\;\sum_{\forall S}\pi_{i,j,k,m,n=1}$$

The CMC model that is used to analyze the proposed system is assumed to be irreducible and has a finite state chain. Accordingly, the CMC is ergodic since it is irreducible and positive-recurrent [23]. Therefore, all the state probabilities π_i,j,k,m,n_ can be derived. A developed Matlab code is used to run all the detailed balance equations and calculate the probabilities of being in any defined state. After finding the probability of each feasible state, the transition matrix Q is derived. This transition matrix contains the transition rates between every two-system state. Having the entire steady-state probabilities π_i,j,k,m,n_ and the transition rates between every two states, the steady-state probability vector π is derived.

### 5.2. Performance Measurements

Using the derived steady-state probability matrix π, we can define and derive the performance measures that can be used to compare and study the QoS metrics for both eMBB and mMTC services, as they have a lower priority than uRLLC. The queuing analysis methods presented in [26,27] were followed. These performance measures include the forced termination probability (PF), the service completion rate (SCR), and the average queuing delay (D). In addition, the system resource utilization for both eMBB and mMTC services is measured. These performance measures are derived and defined as follows:

1. **eMBB PF**: the forced termination probability of the eMBB is the probability that an uRLLC request with higher priority arrives and the in-service eMBB is forced to terminate its in-service connection before its normal end. The in-service eMBB is forced to release its occupied BBUs and assign them to the arriving uRLLC when the system resources are fully utilized, there are no available BUU resources, and the in-service mMTC connections are zero. The released resources are then given to the newly arrived uRLLC. In this case, the forced termination probability can be derived as the average rate of terminated eMBB services divided by the admitted rate of eMBB services. Therefore, we can denote PF_1_ as the forced termination probability of the requests of type eMBB, which can be derived as follows:

(6a) $$PF_{1}=\frac{the\;averge\;rate\;of\;terminated\;eMBB}{the\;eMBB\;admitted\;rate}$$

Using the derived balance equations, the average number of eMBBs forced to terminate is given by:

(6b) $$\sum_{\begin{array}{c} \forall S\\ i+j+k=Candk=0 \end{array}}\frac{\lambda_{1}j}{C-j}\pi_{i,j,k,m,n}$$

The total eMBB admitted rate is designed as $\lambda_{2}\left(1-{PB}_{2}\right)$. Hence, the PF_1_ can be rewritten as:

(7) $$PF_{1}=\sum_{\begin{array}{c} \forall S\\ i+j+k=C\;and\;k=0 \end{array}}\frac{\lambda_{1}j}{\left(C-j\right)\lambda_{2}\left(1-{PB}_{2}\right)}$$

2. **mMTC PF**: This probability can be derived following the procedures used for eMBB. The mMTC is forced to terminate its connection by the arrival of a higher-priority request (in this case, uRLLC and eMBB) when all BBU resources are busy and its queue is full. Similar to (7), the forced termination probability for mMTC is defined as:

(8) $$PF_{2}=\sum_{\begin{array}{c} \forall S\\ i+j+k=C\;and\;n=N \end{array}}\frac{\lambda_{1}k}{\left(C-j\right)\lambda_{3}\left(1-{PB}_{3}\right)}$$

3 The service completion rate (**SCR**): The rate of service completion of both eMBB and mMTC is measured as the total rate of service completion of each type by unit time. Using the derived balance equations, we can derive this rate. Let us assume that SCR_1_ and SCR_2_ are the service completion rates for eMBB and mMTC, respectively. The service completion rate of eMBB and mMTC services is derived as shown in (9) and (10), respectively.

(9) $${SCR}_{1}=\sum_{\forall S}j\mu_{2}\pi_{i,j,k,m,n}$$

This equation represents the service completion rate for eMBB. This rate is computed by adding the service completion rates (departure rate) of all states that represent an eMBB departure, where j represents the number of in-service eMBB, $\mu_{2}$ the service rate of eMBB, and $\pi_{i,j,k,m,n}$ the probability that we are in state (*i*,*j*,*k*,*m*,*n*). Using the steady state characteristics, we can find the competition rate by adding all the states that have an eMBB departure.

(10) $${SCR}_{2}=\sum_{\forall S}k\mu_{3}\pi_{i,j,k,m,n}$$

4 C-RAN resource utilization (**U**) by both eMBB and mMTC services: this metric indicates the efficiency of the proposed system in terms of increasing BBU utilization by allowing both eMBB and mMTC services to be queued and reattempted. Using the system state space S, we can notice that at each state space (*i*,*j*,*k*,*m*, and *n*), the system has C resource units (BBUs), and (*i* + *j* + *k*) BBUs are occupied by either uRLLC, eMBB, or mMTC services. Therefore, the utilization percentage for both eMBB and mMTC requests can be defined as:

(11) $$U=\sum_{\forall S}\frac{i+j+k}{C}\pi_{i,j,k,m,n}$$

5. The average delay (**D**) for both eMBB and mMTC services: each service type has its own queue, and we can consider D as the average delay time for each queued request until it is served. In order to calculate D, we need to know the queue length for both queues 1QeMBB and 2QmMTC. Using Little’s laws, we divide the queue length of each service by its arrival rate. Let us assume that L_1_ and L_2_ are the queue lengths for eMBB and mMTC, respectively.

Therefore, the average delay for eMBB (D_1_) can be calculated as follows:

(12) $$D_{1}=\frac{L_{1}}{\lambda_{2}}$$

where $L_{1}\sum_{\begin{array}{c} \forall S\\ 0\leq m\leq M \end{array}}m\pi_{i,j,k,m,n}$.

In the case of mMTC services, the average delay (D_2_) is calculated as follows: We will include the interrupted connection with its total arrival rate since there will be interrupted requests of type mMTC, shown as feedback, by giving the interrupted request another chance to be serviced in C-RAN. Hence, when we calculate D_2_, we need to add the interrupted connection rate to the mMTC arrival rate. Here, R_Interrupt_ represents the interrupted rate of mMTC. Therefore, D_2_ can be written as:

(13) $$D_{2}=\frac{L_{2}}{\lambda_{3}+R_{Interrup}}$$

where $L_{2}=\sum_{\begin{array}{c} \forall S\\ 0\leq n\leq N \end{array}}n\pi_{i,j,k,m,n}\;and\;R_{Interrup}=\sum_{\begin{array}{c} \forall S\\ i+j+k=C\\ v<C \end{array}}\frac{\lambda_{1}k}{C-v}\pi_{i,j,k,m,n}$.

This equation represents the average delay of mMTC. Using the little theorem, this average can be calculated by dividing the average mMTC queue size (L_2_) by the total mMTC arrival rate. The total arrival rate includes the interrupted mMTC that is inserted back into its queue. Using the system states and the steady state probabilities, this term has the following form:

(14) $$R_{Interrup}=\sum_{\begin{array}{c} \forall S\\ i+j+k=C\\ v<C \end{array}}\frac{\lambda_{1}k}{C-v}\pi_{i,j,k,m,n}$$

The term L_2_ can be found by adding the average number in the queue for all the system states that have queued requests. Using the system states and the steady state probabilities, this term has the following form:

(15) $$L_{2}=\sum_{\begin{array}{c} \forall S\\ 0\leq n\leq N \end{array}}n\pi_{i,j,k,m,n}$$

## 6. Results and Discussion

In this section, the performance measures of the proposed system are studied and explained. We compare the proposed system with other systems, with and without queuing. Following that, we simulate examples of the three methodologies previously mentioned with Matlab and Simulink using Markov chain methods. For both eMBB and mMTC requests, performance is analyzed in terms of their service completion rate (SCR). Other parameters of importance, such as the average delay of both eMBB and mMTC requests and the forced probability, are discussed and analyzed. We assume a single C-RAN with 25 available BUU resources (i.e., C = 25). The arrival rate of all the requests to the C-RAN is classified into three priority-based 5G use cases, including uRLLC, eMBB, and mMTC, respectively. We assume that, unless otherwise specified, uRLLC, eMBB, and mMTC have the following arrival rates: λ_1_ = 1, λ_2_ = 1, λ_3_ = 2, respectively. Their service rates are assumed to be μ_1_ = 0.5, μ_2_ = 1.0, and μ_3_ = 1.0 for uRLLC and eMBB, respectively. Additionally, we assume that the sizes of 1QeMBB and 2QmMTC are 4 (M = 4) and 2 (N = 2), respectively.

**Figure 4:**
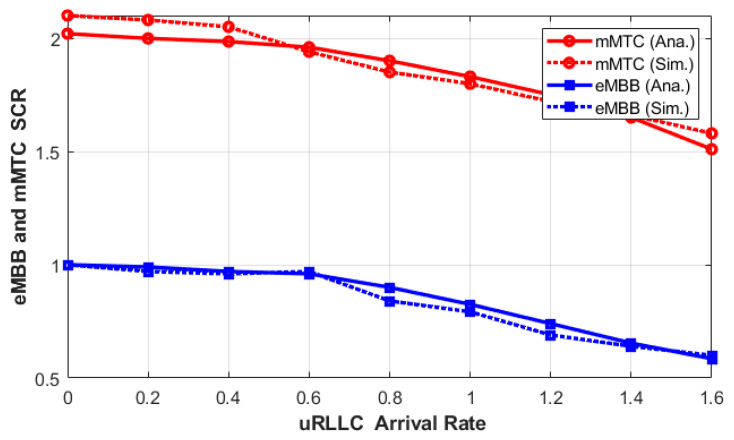
Analytical and simulated results for eMBB and mMTC SCR as a function of uRLLC arrival rate—Methodology III.

First, the analytical and simulation results are compared and validated using the above parameters. In Figure 4, we validated the results of the system using Methodology III. As it is depicted in the figure, the behaviors of both analytical and simulation results for the eMBB and mMTC’s SCR as a function of the uRLLC arrival rate are plotted, and the results are a good match. Furthermore, we can see from Figure 4 that the analytical and simulated SCR results are closer for eMBB than for mMTC when the arrival rate of uRLLC is low. The reason for SCR variation is that, in the 2QmMTC, there are two queuing processes during the arrival and the interruption rate, and the interruption rate is uncertain. In the case of the 1QeMBB queue, the arrival rate is Poisson. Thus, the proposed scheme shows acceptable matching between both simulation and analytical results.

**Figure 5:**
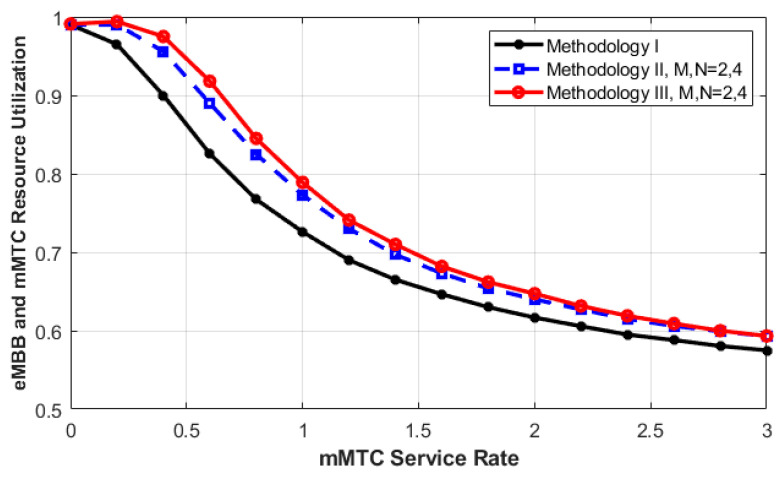
eMBB and mMTC resource utilization as a function of mMTC service rate for different methodologies.

Using all three methodologies, the combined resource utilization of both eMBB and mMTC services has been explored. The service rate of mMTC is variable, while it is fixed for uRLLC and eMBB. As depicted in Figure 5, the resource utilization of both eMBB and mMTC services decreases as the service rate of mMTC increases. This is because when the service rate of mMTC increases rapidly in a short time, more in-service mMTC requests complete their service more rapidly. In this case, the probability of keeping the resources busy with the mMTC requests decreases. Moreover, since the arrival rates of uRLLC and eMBB requests are fixed in this case while the service rate of mMTC increases, the probability of a new arrival requesting an idle resource in the other two cases decreases.

Therefore, we can see that the resources in the C-RAN will stay idle for a relatively longer time. This makes sense when the resources are not greatly utilized in the system. Consequently, increasing the service time of one type while fixing it for others results in a reduction in resource utilization. Therefore, using the queuing concept allows the arrived eMBB and mMTC requests to be queued and the interrupted mMTC request to be queued again (as employed in Methodologies I and II). This process increases resource utilization as more chances are offered to the queued requests (including the interrupted mMTC requests) to reattempt the service again in the C-RAN.

**Figure 6:**
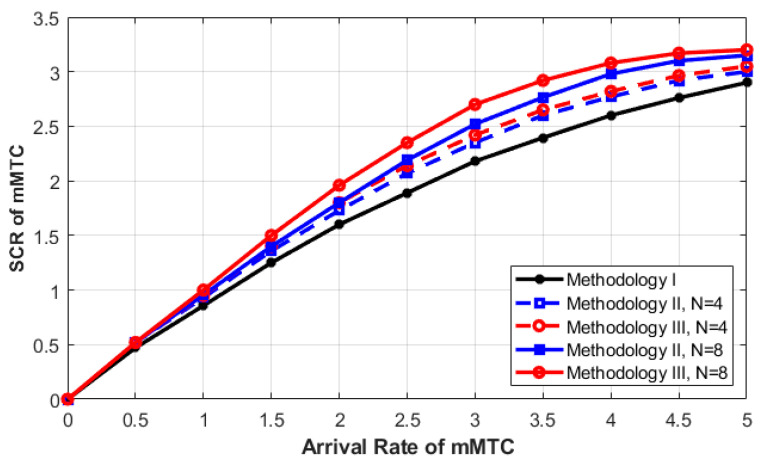
**mMTC** SCR as a function of the **mMTC** arrival rate.

Figure 6 shows the SCR of mMTC service versus its arrival rate using all three methodologies. The arrival rates of both uRLLC and eMBB are set to a fixed value. As can be seen from the figure, the SCR of mMTC increases rapidly, then tends to move smoothly to a peak value in the sequence. This is because when the arrival rate of mMTC increases at the beginning, the SCR also incrementally increases. After that, the SCR curves move into a peak or max value because all the C-RAN resources have started to be completely utilized. Further, we can observe from the figure that, in addition to the queuing model, increasing the queue size will improve the SCR of the mMTC service. When we increase the queue size from 4 to 8, the curve of the SCR shows better improvements. Therefore, increasing the queue size allows extra requests to potentially be queued. Since the requests of uRLLC have a fixed rate and those of mMTC have an increasing rate, this leads to more queued mMTC requests and a higher probability that mMTC services will complete their service connection. Hence, the SCR increases by increasing the queue size. Now, comparing the SCR using Methodologies II and III, it can be seen that Methodology III results in a higher SCR as compared with Methodologies II with no feedback link for interrupted mMTC requests.

In regards to Methodology II, all interrupted mMTC requests will be forced to terminate the service even if the 2QmMTC has available resources, and interrupted requests will be ignored during SCR calculations. However, the SCR behavior in Methodology III is improved as compared with that in Methodology II because the in-service mMTC requests that have been interrupted are given a second chance to join the queue and reattempt accessing resources. With this feedback link, Methodology III outperforms other methodologies in terms of SCR rate.

**Figure 7:**
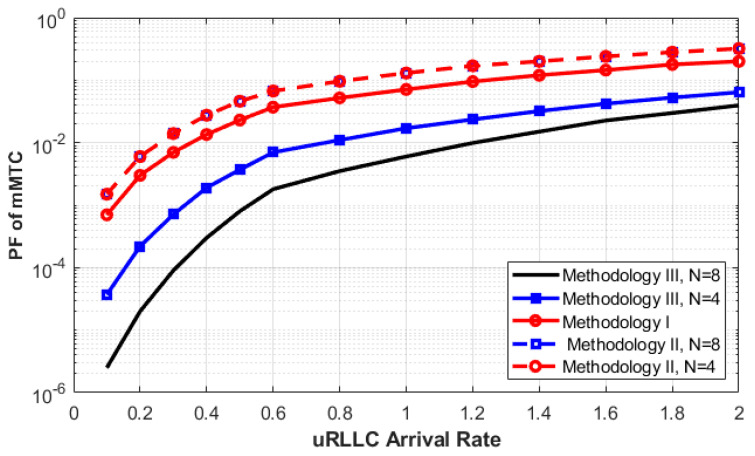
**mMTC** PF as a function of the **uRLLC** arrival rate for various methodologies.

In Figure 7, the PF of mMTC requests is depicted against the arrival rate of uRLLC. The main goal of this figure is to explain the effectiveness of Methodologies II and III when the arrival rate of the uRLLC request with the highest priority increases. As we can see from the figure, as the arrival rate of uRLLC requests increases, the PF for mMTC requests increases using all methodologies. The reason behind this behavior is that when eMBB and mMTC requests are forced to terminate by the higher priority slice, the arrival rate of uRLLC keeps increasing, thus increasing the usage of C-RAN resources. When the system accepts more uRLLC requests, many in-service eMBB or mMTC requests will be interrupted, and most of the BBUs will have been taken by the arrival of uRLLC requests.

In addition, the PF of the mMTC requests does not improve much with or without using two queues (Methodology II). The reason is that when we allow the eMBB and mMTC requests to be queued, the interaction between them and the uRLLC request will be greater than when we have no queues. This interaction will result in an increase in the calculated PF.

Therefore, allowing the arrival request of mMTC to be queued is not enough, and an effective method should be used to efficiently minimize the PF of mMTC. Methodology III is proposed to solve this issue by allowing all requests that have been interrupted to rejoin the queue while there are free spaces in it and it is not full. In addition, increasing the queue size to 2QmMTC can also minimize the PF. Finally, it is clear that the idea of Methodology III can be selected as a better solution for having a lower PF.

**Figure 8:**
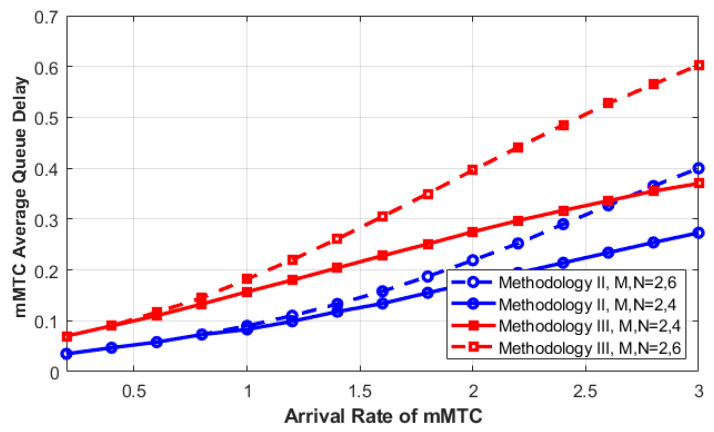
**mMTC** average queuing delay as a function of **mMTC** arrival rate for various methodologies.

As illustrated in Figure 8, the average queuing delay of mMTC requests for both Methodology II and III is studied and compared. As we can see, the performance of Methodology II outperforms that of Methodology III in terms of average queuing delay. The reason for this is that when we allow the interrupted mMTC request to rejoin its queue, the average queue size will increase. Increasing the number of queued requests will increase the average queuing delay, as the queue length is directly proportional to the average delay as derived in the analytical model. Therefore, Methodology III reduces the PF of mMTC requests and increases their average queuing time. Therefore, there is a tradeoff between increasing the average delay and decreasing the PF for mMTC requests. Since the mMTC is a delay-tolerant service, decreasing the PF has more benefit in terms of SCR than decreasing the average queuing delay. The best solution for this conflict is to find the optimal value for the queue size that can balance these two conflicting factors.

To summarize, the main goals of the proposed system have been met. It proved that using the queuing system with feedback can improve the overall utilization of C-RAN resources (BBUs) and the SCR of both lower slice types. As we increase the queue size, the forced termination probability can be improved further at the expense of increasing their average queuing delay. The size of these queues can be designed according to the maximum acceptable delay required by each traffic slice type.

## 7. Conclusions and Future Scope

In this research, we propose a priority-based resource allocation for 5G C-RAN with multi-traffic slices categorized based on the slice’s traffic priority and QoS. It includes two queues for lower-priority traffic, eMBB and mMTC, to increase their chances of completing their services. Hence, this will increase the utilization of C-RAN resources without degrading the QoS of the highest priority class. An analytical model using CMTC with multidimensional states is developed to derive the system performance measures. The analytical results of the proposed system are conducted and validated with simulation-based results. In order to investigate and compare our proposed system, it was studied using varied methodologies. In Methodology I, the system operates without using queues for lower-priority slice types, similar to the previous related models, and is used as a reference methodology. In methodology II, we use two queues for both eMBB and mMTC without allowing the interrupted mMTC to rejoin its queue for a possible service reattempt. Finally, Methodology III is similar to Methodology II in that it allows the interrupted mMTC to rejoin its queue for a possible service reconnect.

Based on the conducted results, Methodology II and Methodology III show differences in behavior when an mMTC is interrupted. With Methodology II, when there are few requests of type uRLLC and many requests of type eMBB and mMTC, the model is very efficient in terms of eMBB and mMTC SCR and forced probabilities. Methodology III allows the interrupted mMTC to rejoin its queue and hence reduces its forced termination probability, while Methodology II leads to its increase. Therefore, Methodology III will work well when we have high arrival rates of uRLLC, while Methodology II is better when we have low loads of uRLLC and high loads of mMTC. Nevertheless, Methodology III is very active in the sense that uRLLC is very active.

In the future, a mixing of these methodologies could be very effective if they are made hybrid by adding multiple constraints and utilizing multi-objective functions that may enhance the effectiveness of the future schemes proposed.

## Figures and Tables

**Table 1:** 5G use cases and their priorities.

Case	Ultra-Reliable and Low-Latency Communications (uRLLC)	Enhanced Mobile Broadband (eMBB)	Massive Machine-Type Communications (mMTC)
Priority	1	2	3
Min. bandwidth	25 Mbps “Low”	100 Mbps “High”	100 Kbps “Low”
Latency	Low	Medium	High
End-to-end latency	1 ms	10 ms	s to h

**Table 2:** The pseudocode (algorithm) of the proposed scheme.

uuRLLC Arrival Process: **IF** available BBUs > 0 **THEN** Accept uRLLC **ELSE** **IF** No. of eMBB or mMTC in progress > 0 **THEN** **DO one BBU Preemption:** {in-service mMTC is selected first if any} **- IF** interrupted service == mMTC **IF** mMTC queue is not Full Insert mMTC in its queue **ELSE** mMTC Leaves the system } **- ELSE** eMBB Leaves the system **-END** Assign the uRLLC to released BBU **ELSE** Block uRLLC **END** **END**	eMBB or mMTC Arrival Process: **Case of eMBB:** **IF** available BBUs > 0 **THEN** Accept the request eMBB **ELSE** Check eMBB queue **IF** eMBB queue is not full **THEN** Insert eMBB in its queue **ELSE** Block eMBB **END** **END** Case of mMTC: **IF** available BBUs > 0 **THEN** Accept the request mMTC **ELSE** Check mMTC queue **IF** mMTC queue is not full **THEN** Insert mMTC in its queue **ELSE** Block mMTC **END** **END**

**Table 3:** Parameter descriptions.

Symbol	Description
uRLLC	ultra-reliable low-latency communications
eMBB	enhanced mobile broadband
mMTC	massive machine-type communications
C	total number of LTE-A RBs
λ_i_	the arrival rate of service request i
μ_i_	service rate for i service request
1QeMBB	queue of eMBB
2QmMTC	queue of mMTC
M	size of 1QeMBB
N	size of 2QmMTC
PF	forced termination probability
BP	eMBB, mMTC blocking probability
i	number of ongoing uRLLC services
j	number of ongoing eMBB services
k	number of ongoing mMTC services
m	number of queued eMBBs in 1QeMBB
n	number of queued mMTCs in 2QmMTC
D	eMBB and mMTC average delay time
U	eMBB and mMTC resource utilization

**Table 4:** Boolean indicators.

Boolean Indicators	Condition	Boolean Indicators	Condition
***α_1_***	*1, for i < C* *0, for i = 0*	***α_6_***	*1, for m > 0* *0, for m ≤ 0*
***α_2_***	*1, for k = 0* *0, for k <> 0*	***α_7_***	*1, for n > 0* *0, for n ≤ 0*
***α_3_***	*1, for i > 0* *0, for I = 0*	***α_8_***	*1, for m < M* *0, for m = M*
***α_4_***	*1, for k > 0* *0, for k ≤ 0*	***α_9_***	*1, for n < N* *0, for n = N*
***α_5_***	*1, for j > 0* *0, for i ≤ 0*		

## Data Availability

Not applicable.
